# Collectanea Medica, Consisting of Anecdotes, Facts, Extracts, Illustrations, Queries, Suggestions, &c.

**Published:** 1818-06

**Authors:** 


### COLLECTANEA MEDICA,

CONSISTING OF

### ANECDOTES, FACTS, EXTRACTS, ILLUSTRATIONS, QUERIES, SUGGESTIONS, &c.

RELATING TO THE

*History or the Art of Medicine, and the Auxiliary Sciences.*

Quicquid agunt medici,
nostri farrago libelli.

*Is it impossible to free the Atmosphere of London, in a very considerable degree, from the Smoke and Deleterious Vapours with which it is hourly impregnated?* By WILLIAM FREND, Esq. late of Jesus College, Cambridge, and Actuary of the Rock Insurance Company.

THE smoke of London, first viewed from a distance, affords a sight which strikes a foreigner with astonishment. The inhabitants are so accustomed to it, that they cease to regard it as a matter of much inconvenience, though a box of clothes, sent from the metropolis, sensibly affects their country friends. In fact, the Londoner is in the situation of a man who lives in a smoky house: he is used to it: but every one who comes to visit him, perceives the difference between his sooty chambers and a well-ventilated mansion.

There are people, who, from long habit, are not disgusted with dirt and nastiness. The Hottentot returns to his grease: but, I believe that, if the smoky atmosphere of this great city was exchanged for a purer air, none of the inhabitants, or the occasional visitors of the metropolis, would lament the loss of their black fog. All talk of the smoky town; but they sit contented under their dense vapours, from an opinion that the evil is incurable.

This is the case with every improvement that is suggested. A few years back, the visitors of Bedlam were assured that it was impossible to keep the unhappy patients but by such immense locks, bolts, and chains, as shocked humanity. The very people who were thus manacled and fettered, are now walking about at their ease in the New Bedlam, and are more tractable in consequence of the removal of these restraints. I went over this excellent institution the other day, and, on contrasting it with what I had seen in the former mansion, could not but congratulate human nature on the modern improvements in the treatment of the greatest calamity to which it is liable. The application of the same common sense would produce similar improvements in a variety of instances.

To form a judgment of the smoke of London, a person should take a few turns on Blackfriars' Bridge, in the middle of the day, and notice the effect of the vomitories of smoke that surround him. A similar walk, at night, will point out more strongly the defect in their construction, when he perceives, from many of them, volumes of flame issuing forth, as from so many volcanoes. If he takes his morning walk in the middle of summer, the contrast between the smoke from the furnaces, and from the ordinary fires, will be particularly striking. I conjecture, that the smoke, occasioned by a vast variety of manufactories, forges, glass-houses, &c., is at least three times as great as that proceeding from all the chimnies for family use: and it is to be recollected, that from some of these furnaces issue forth matter of a most deleterious nature.

On the mischief arising from noxious vapours, the medical men are the best judges; but it is somewhat remarkable, that so little attention is paid to this subject by the legislature. Within these few years, an instance occurred, which brought it forcibly within my notice. There was a small green lane near me, which I used to frequent as a walk before breakfast. Soon after I had discovered this little walk, preparations were made for building, and, in a short time, two spacious erections appeared, both furnished with furnaces, the chimneys of which were not carried ten feet above the roof. One of them was applied to the smelting of lead, the other to the melting, I think, of iron. In a short time proceeded the usual appearances of such works, and the air was proportionably infected. A great road, which promises to be one of the most considerable thoroughfares in London, has lately been made near this place; and, when the wind is favourable, the pale whitish smoke from the lead manufactory is sensibly felt by those whom business or pleasure carries that way. On the erection of these buildings, I could not help pointing out to the owner the high chimney of the great manufactory near the Asylum, and with what advantage, both to himself and to the public, the very meritorious engineer, the owner of them, had constructed his works. But how could it be expected, that such an expense should be incurred, since the public did not require it: and all that was wanted, in this instance, was a common blast furnace, whose smoke, when it reached the top of the works, was of no farther concern to the owner.

Common sense ought to have instructed us, that an individual, who makes a fortune by his works, ought to take care, that it should be done at the least inconvenience to his neighbours. But, in the case of the great works in and about London, this principle is almost entirely neglected. A man, for example, erects a brewhouse upon a small scale: his business extends, and with it his works gradually increase, so that what was at first scarcely felt, now becomes a nuisance to a very great extent, and a volume of smoke is emitted, which is felt in magnificent houses at a very considerable distance from his brewery. It will be asked, how can this be remedied? To this may be replied, let a man on

Blackfriars' Bridge look around him, and observe the state of the different manufactories. He will see from one a large column of smoke, driving before the wind, scarcely impaired, to a great distance; and from another a much smaller column, much sooner dissipated, and yet, perhaps, the coals consumed in the latter are ten times as much as in the former. Whence, then, is this difference? It is in the height of the chimneys; and common sense, therefore, points out that the height of the chimneys should bear some proportion to the quantity of coals consumed in the furnaces beneath them.

It is possible to contrive it so, that the column of smoke proceeding from the greatest vomitory in the metropolis shall not exceed that from a common kitchen chimney. To do this, it is necessary only to have a proportionate length of chimney. Suppose a brewhouse to be constructed in a square, the sides being a hundred feet in length, and the height of the building to be seventy feet. Let the furnace be near one of the sides, communicating its smoke to a chimney, inclosed at top, twelve feet square at the bottom, and decreasing in dimensions to half the height of the building. At half of the height of this chimney let there be two apertures, one on each side, and a flue from each to run round the building till it comes to the top. Supposing each to make a complete turn, its smoke must pass through above four hundred feet, and let it then pass through the vent at the top, thirty feet high. Thus the column, which now bursts forth in such a torrent, will be diminished more than one half at the lower aperture of the flues, and each separate column, thus formed, will be diminished in a very great proportion by the quantity of space it has to go over. This quantity will be greatly diminished by chambers being formed at each corner of the building, similar to that of the chimney first erected; and, besides, instead of coming out at top at one hole, the upper chimney may be so contrived, that there shall be several apertures, and the smoke at last will be speedily diluted with the common air.

This, it will be said, is all very good in theory, but it will not do in practice. I went over, a few years ago, the works of some celebrated mines in the north. They give support to a considerable population in the neighbouring village. This population had been subjected to much disease from the effluvia of the lead in its processes of smelting. The proprietors made an alteration in their works. Their vast furnaces were now so constructed, that the smoke from them was conveyed to a common chimney, and this chimney was constructed under ground in the rising hill, the vent being at a considerable distance from the works. The consequence was, that a vast quantity of deleterious matter was retained in the chimney, and afforded an annual rent to the proprietors; and what came out at last was at such a distance from the workmen, that it could not produce any injurious effects to their health. The village now performs its daily task with alacrity; and, I believe, that that which used formerly to be so dangerous an occupation, is now more innocent than a vast variety of employments in the metropolis.

I lay it down, then, as a position, that the smoke, emitted by the vomitories, may be greatly reduced in quantity; that the expense of doing it is trifling in comparison of the profits of the works; and that the legislature will not be ill employed in taking such measures as shall secure the public from its present nuisance.

If it be desirable that the trial should be made, the first thing seems to be, to inquire into the present state of the buildings employing furnaces, comparing each with the other, according to the quantity of coal consumed; to examine into the expense that would be incurred by conducting the smoke in the manner above recommended, or something similar to it, according to the nature of the works; to ascertain, whether the proprietors of old works should be put wholly to this expense, or be indemnified in part by the public; and, lastly, to prevent any new works, unless on regulations adopted on the principle that a furnace should not be permitted to emit more smoke than a common chimney. If the last principle be adopted, the erection of a very extensive manufactory will not produce a hundredth part of the smoke that would be occasioned by ordinary houses, built on the same extent of ground.

These hints are thrown out with a view of exciting some persons to take this matter into consideration. A few copies only have been printed for the use of the writer's friends, and for some members of the legislature, to whom he has taken the liberty of submitting them. They are merely hints; but, if a committee of the legislature should be appointed to examine this subject, he has no doubt, that the light thrown upon it by engineers and manufacturers will shew that the diminution of the smoke of London will be highly beneficial both to the proprietors of works and to the public.

(Signed) Wm. FREND.

---

[As it is of the utmost importance that a medical record should be preserved of all the symptoms of Miss M'Avoy's extraordinary case, we transcribe the following from the Monthly Magazine of the last month.]

*Liverpool; April* 16, 1818.

Sir,—In your letter of the 8th of April, you have requested further information respecting *the case and question of Miss M'Avoy*. On the 19th of November last, I stated to you, that Miss M'Avoy, after suffering from great pain in the head, producing giddiness, &c. had, on the 15th of that month, been seized with convulsions: they continued almost incessantly, and with considerable violence, until the 25th of November. On that morning a discharge took place of fluid from the head,—similar to that which had occurred in June 1816, but in smaller quantity; an account of which is given in the Narrative. After this discharge the convulsions continued with less violence, during that day and the 26th,—when they ceased in the day, but occasionally occurred during the night, until the 29th of December. They were followed by the convulsive motion of the diaphragm, difficulty of breathing, and oppression about the region of the heart. The right side was paralytic, and the peculiar sensibility of the right foot, upon any sudden pressure, was remarkably evident. On the 17th of January, the suspension of breathing came on, and increased so, as sometimes to be suspended four times in the space of five minutes, for about ten seconds each time. During the intervals, the convulsive motion of the diaphragm, &c. returned, and ceased upon the suspension again coming on. When she lies upon her back, with the back of her head upon the pillow, she feels a sensation as if she were sinking through the bed and floor: if she lie on either side, the most horrid dreams assail her; and she is much troubled with night-mare. On the 31st of March, in consequence of her little brother being seized, whilst lying upon the sofa with Miss M'Avoy, with a rattling noise in his throat threatening suffocation, from the bursting of an abscess internally; she fell into convulsions, which come on during the day very frequently, but still oftener in the night. These are now attended with the suspension of breathing, which is of much longer duration than formerly. Some time ago, it was observed, that a considerable protrusion of the ribs on the left side had taken place, attended with extreme pain on pressure. The swelling has since very much increased, and seems to extend to the cartilago ensiformis, over a great part of the epigastric region, into the left hypochondrium; and along the whole course of the spurious ribs. It is not protruded as before upon any particular point of the ribs, but seems to be a generally increasing swelling, acutely painful upon the least pressure with the fingers. The pulse has been generally between 96 and 108 pulsations in the minute; sometimes much slower, and sometimes more frequent. By Fahrenheit's thermometer, the heat of her hands was 86, in the mouth 106, and in the upper part of the eye-lid 102 degrees. She obtains very little sleep, that she is aware of. Her bowels are very often costive, although less so than formerly, and she takes very little food. About a month ago, the whole food she took in one fortnight was only half a pint of coffee, milk, and sugar. The bowels were not opened during this time; and, in four or five days, she only passed about half a pint of urine. She was more emaciated during this period than at any time during her illness. The menses appeared on the 19th of November, and continued for a few hours; but since that day they have not recurred. The paralytic affection, and the peculiar sensibility of the right foot, are removed; but, unlike what has occurred before, she cannot support herself in an erect posture, but is obliged to be carried, if removed from the sofa. She employs herself, when able, in sewing, netting purses, and making little ornaments with coloured papers and silks. She has now and then been able to name a colour; but, since this illness commenced, with no degree of certainty, and she was not acute in this respect for some time before the convulsions came on. When making use of the coloured papers, she was asked how she knew the colours, which consisted of red, green, and yellow, and common writing paper: she said, it was not the colour, but the peculiar texture of the paper by which she knew the colour. The mode she adopted to know the colour of the silks, was by winding them round pieces of card, variously formed by herself; and, by this means, she recollected which card contained the one silk, and which the other. Four or five days after she had finished the purse, she had been so unwell, as scarcely to be able to do any thing; and, upon giving one after the other four of the cards containing the silks, she only named one of them, having apparently forgotten the form of the card which contained the particular silk. She was uncovered when desired to tell the time of the day or any colour, in which she was almost uniformly unsuccessful. I have now given you a general outline of Miss M'Avoy's case, from the 15th of November last to the present day, extracted from the intended Continuation of the Narrative.

You ask me also respecting the state of the question about Miss M'Avoy. As I presume you allude to the report which has been very industriously circulated in almost every quarter, through the medium of newspapers, the Medical Repository, and some other monthly publications, and Mr. Sandars's Pamphlet, of Miss M'Avoy being an impostor, I can only say, in answer, that during a very long attendance, and from various experiments, I am convinced that she is no impostor, but an amiable, well-disposed, unoffending female; who, from the peculiar nature of the powers she possessed, has subjected herself to the animadversion of individuals; some of whom, I have no doubt, are guided by pure, but mistaken principles; but the greater part, I have reason to believe, have been influenced by motives which would not bear the test of scrutiny.

It is not my intention, at present, to enter into a discussion of the merits or the demerits of Miss M'Avoy's antagonists. If the fact of her blindness be proved, all the objections against her will fall to the ground; and, as far as I can judge, I am satisfied of it, and in this opinion I am supported by those medical gentlemen who have paid the greatest attention to her case.

On the other hand, the supporters of the opinion that she can see, are generally derived from that class of individuals who have had the slightest, if any, opportunity of examining into the state of her eyes. Indeed, some of these gentlemen go further, even into the regions of romance, and give her credit for more acuteness of sight than any other person; but this opinion only rests upon mere supposition, not having the least shadow of probability. What has struck me as very peculiar in the conduct of the antagonists to Miss M'Avoy's integrity, is the mode they have adopted of disseminating their opinions, by assuming as facts what they must, in many instances, be aware are not so, and by a reference to Mr. Sandars's pamphlet, as a work which has proved every thing against her. Those who are aware of the motives which brought forward this publication, do not rely upon the proofs Mr. Sandars has addressed; and when his facts are compared with those related in the Narrative, it will be found, he has only taken the arguments on one side of the question, without bringing forward any in her favour: a mode not the best adapted for the discovery of the truth, or for convincing the minds of the unprejudiced. The present state of health of Miss M'Avoy is so very precarious, that a very short time may determine whether she will recover the power or lose it altogether, or whether her life may be continued for any considerable period. If either the former or the latter event takes place, it may enable us to obtain the truth by further experiment, or by actual examination into the cause of her disease; and, whenever the result of these experiments, or that examination may be, the public shall immediately be informed of it through the medium of the different periodical publications.

I have the honour to be, Sir, your very obedient servant,

*To Sir R. Phillips, &c. &c. London.* M. RENWICK.

---

[From our Correspondent at Paris.]

[We transcribe the following case of pseudo-syphilis, or cachexia syphiloidea, or any other term our readers please, from the *Journal Universal des Science Medicale* for March last. Not that the case is particularly new, but it serves to show how much the patients of our continental neighbours suffer from the ignorance of their surgeons of the Hunterian doctrine.]

*Observations on Serious Accidents arising from an inconsiderate use of Caustics and Mercurials, in a Case of Syphilitic Inflammation.* By Dr. JANIN.

M. B. æt. 45, of a lymphatic temperament, living at Paris, contracted, at the latter end of August 1814, a syphilitic blennorhagia. In the first days' heat and pain on making water; then swelling and redness of the urethra; a slight limpid discharge, and appearance of a chancre on the upper part of the crown of the gland. A surgeon prescribed a nitrous beverage, and cauterized the chancre with a solution of argentum nitratum. A few days after, considerable swelling of the penis. The patient, whose business obliged him to go out, walked nearly six miles, and returned home much fatigued: the swelling of the penis increased. The next day phymosis: great difficulty and pain in making urine: baths and emollient fomentations gave no relief.

The surgeon divided the prepuce in the upper part, but merely half its length, which was insufficient to stop the rapid progress of the inflammatory symptoms. He administered, internally, Van Sweeten's solution of sublimate; and divided the remainder of the prepuce.

The chancre made a rapid progress: it was dressed with double mercurial ointment: excruciating pain followed; want of rest, and a mortification of the part. The chancres increased, which, cauterized with sulphat of copper, soon extended as far as the pubis, after covering all the left side of the penis. The wound became grey, very painful, and gave a fœtid ichor. It was dressed with yellow basilicon, mixed with the red oxide of mercury: this was followed with insupportable pain; the teguments which covered the pubis separated and came off, and the subjacent cellular tissue was in a great measure destroyed.

The patient consulted another surgeon, who insisted on the internal and external use of mercurials. The violence of the pain prevented the patient from taking any sleep: he became extremely meagre, and was afflicted with diarrhœa, accompanied with colics.

Called in on the 17th of October, 1814, and found the patient in the following state:

The chancre had destroyed all the left side of the penis, and a part of the teguments covering the pubis; the *corpus cavernosum*, and the cord of the spermatic vessels, on this side, were perfectly dissected, and the lower part of the symphisis of the pubis was naked. On raising the teguments, slightly, there was perceived, in the midst of a very fetid suppuration, some of the fibres of the pyramidal muscle. There was below the gland a very hard swelling of the cellular texture; the canal of the urethra was open immediately below the symphisis of the pubis, in the triangular space formed by these bones, and, by parting the *corpus cavernosum*, the urine issued entirely by this opening.

The first thing to be done was to calm the excruciating agony suffered by the patient. I resolved on employing opium externally and internally. I covered the parts affected with lint steeped in a decoction of linseed, with a small compound wine of opium. This having given only little relief, I thought that the wine of opium might have irritated it, and substituted the gummy extract. I administered also two grains daily to the patient internally.

At the end of a few days the wound appeared sensibly to improve; the pains were less violent; he got a few hours' sleep; the ulcers became red, and cleaner, instead of being grey, as before. I then attempted, but in vain, to pass the sound, and therefore resolved to wait, and ordered a sudorific beverage of sarsaparilla, guiacum, and sassafras.

The wound continued to mend, and the patient recovered his sleep and strength gradually. The cicatrix produced a shortening of the penis towards the pubis; and there still remains, at the root of the penis, on the left side, a fistula, for which he is obliged to use elastic bougies. Janin.

---

### *Extract of a Letter from M. Vicat to the Editors, on a Worm voided from the Abdomen of a Spider.*

One day in the present month of August, I perceived a large spider, which had concealed itself in one of the blinds of my window: I dragged it gently with a piece of stick on to a small board, which I covered with a glass. This spider, as far as I could judge, seemed to belong to the species commonly called *garden spiders:* the legs and body were smooth, demi-transparent, of a reddish white, and without streaks; the belly of the size of a small nut, exceedingly stretched, without hair or down, and of an olive colour. I put several flies under the glass, the buzzing of which seemed to disturb it; it then drew itself up, by drawing in its legs, but without forming into a ball: when a fly came too near, it put it aside, but without showing any hostile intention. Vexed at its indolence, I gave it a bee for a companion, and then a grasshopper; the latter seemed at first to tire it much by its leaps and petulance; but, at length, it became insensible to all these vexations: the flies themselves passed with impunity over it without its paying any attention to them. Despairing now of seeing any battle as I had expected, I was going to throw the whole out of the window, when I perceived a very thin white point come out of the belly of the spider, lengthen itself, roll in a spiral form—rings on rings; while, at the same time, the belly of the spider relaxed by degrees, and became empty and wrinkled like a burst bladder. I soon perceived a worm of a dirty white, from about a millimetre in thickness, to two decimeters in length, which, resting on a bundle of rings, raised and darted here and there a slender point, which seemed to seek for support. I put this worm in fresh water, where he has lived for more than a fortnight quite well: he sometimes unfolds the whole extent of his body, then draws himself up quickly, and entangles his rings in such a way that it is difficult to conceive how he can afterwards unfold them.

The spider died during this sort of delivery. I am too ignorant of natural history to engage in the least discussion on this phenomenon; but I warrant the exactness of the fact I have just described. I will send the worm to any naturalist who would wish to examine it.

*Souillac; Sept.* 17, 1817.

*Further Account.*—I have observed this worm attentively, from the time of its escape to the instant of my departure for Paris,—that is to say, for two months and a half. He moved about a great deal at first; and seemed to entangle his rings on purpose to unfold them afterwards: I began to understand the object of this exertion, by observing a very thin transparent pellicle, in the form of a gut or sheath, of which he freed himself entirely: in a word, he left his first skin. I perceived at the same time, nearly in the middle of his length, a solution of continuity which I can only compare to a bubble of air interposed between two columns of air in a barometer; supposing the tube to represent the skin, and the mercury the opaque matter which constitutes the substance of the worm. This discontinuity did not destroy in any manner the dependance which I always observed between the motions of the extreme parts,—that is to say, for example, if the head began with an undulation, it was propagated to the tail, and so with the other motions.

On getting near the month of December, the worm remained, for several days together, rolled up and immoveable, which I attribute to the cooling of the water in which he lives. But, what is remarkable, he suddenly came out of this species of lethargy, and moved with great vivacity, when I directed a solar ray on the vessel which contains him, and at the very instant when the ray touched him.

The worm found in the belly of the spider is a species of *filaria*. Several insects, particularly grasshoppers, often have the like. M. Latreille extracted one from the abdomen of a *phalangium*, an animal very near the spider; but we do not know if it had been observed that this sort of worms can live in water.

---

*Poisoning produced by a Decoction of Black Hellebore; communicated to the Medical Society of Emulation.* By FERRARY, Druggist at Saint-Brieux.

A FARMER's servant having felt unwell for two or three months past, went to consult a *healer* of the name of Pierre Sanneguy, called *Le Mouton*, who lived four leagues off: he got three substances from him, which were discovered to be,—one, the root of Solomon's seal, (*convallaria polygonitum*, L.); the second, leaves of ground ivy, (*glecoma hederacea*, L.); and the third appeared to be the root of black hellebore, (*helleborus niger*, L.) The patient boiled these ingredients in cider, till a quart of liquid was evaporated. He drank a glass full; and, from curiosity, his master took a similar dose. Three quarters of an hour after, they began to feel violent colics; nevertheless, the servant thought he must take another dose like the first: the symptoms then became more violent; vomiting, followed by delirium, and violent convulsions, attended with excessive cold, which nothing could diminish, produced death in less than two hours.

The symptoms of the master were as alarming, only less rapid; however he expired three quarters of an hour after his servant.

The magistrates, informed of this event, which had already happened three times in one year, charged Mr. Ferrary, jointly with a physician and a surgeon, to obtain all the necessary information on the spot; and to ascertain, by the examination of the bodies, the cause of the death of these individuals.

The bodies were opened, sixteen hours after death: the same alterations were found in both; but much more remarkable in the servant.

"The lungs were gorged with blood; the mucus membrane of the stomach was in a considerable state of inflammation, of a blackish-brown colour, and reduced almost to a gangrenous state. Nothing remarkable was found in the œsophagus, or in the intestines."

I shall not dwell on the details of the chemical investigations made by the medical men on the matters contained in the organs of these two victims of quackery. As the substance was vegetable matter slightly characterised, little information was to be expected from chemical analysis; but what was more important, all that had preceded the poisoning was well known; also how the drink had been prepared, and the symptoms which had come on immediately after it was swallowed. The violence of these symptoms had been in proportion to the doses of the preparation: in short, a portion still remained, which could be tried by experiments on animals,—and this was done. Two ounces of the mixture found in the stomach of the two men having been given to a cat, the animal made the most violent efforts to vomit, fell into a state of extreme weakness and insensibility, and died in the twenty-four hours.

Messrs. Hipp, Cloquet, and Cavinton, charged by the Society of Emulation to give them an account of this communication, after having discussed the opinions of Mr. Ferrary, relate an experiment which they tried themselves, and which confirms the conclusions of the druggist of St. Brieux on the venomous properties of the root of hellebore. These gentlemen gave to a dog, of a moderate size, and very robust, eight ounces of a decoction made with cider and an ounce of the root of black hellebore in powder; the action of the poison was evident at the same moment: the animal became immoveable, the circulation seemed at first slackened, then quickened; the motion afterwards returned, but only in the limbs, while the trunk of the animal resembled a lumpish mass, to which animated parts were attached; his fore and hind legs were stiff alternately; the tail was also in almost continual motion; and the head, after long efforts, was at last thrown backwards on the back. Notwithstanding his continual efforts to vomit, the animal could only succeed once; his contortions sufficiently showed the pains to which he was a prey: in fine, he expired twenty minutes after the administration of the poison.

On opening his body immediately, the whole digestive canal was found inflamed, from the end of the œsophagus to the intestinum rectum; the stomach was distended and filled with a great quantity of a mixture of bones, meat, and a part of the liquid poison not yet absorbed; the folds of the mucus membrane of that viscus were in a very intense state of inflammation, and of a crimson red colour, but without any trace of corrosion. The duodenum was in the same state; while the pyloric extremity of the stomach presented traces of inflammation much less lively. The rest of the intestinal canal partook of this inflammation, which went on diminishing down to the rectum; the bladder contained no urine; its internal membrane was thickened and red. The organs of the chest only exhibited a slight sanguine obstruction.

These facts, which agree with the experiments previously published by Orfila, on the venomous action of hellebore, hardly allow us to doubt that the death of the two individuals was occasioned by that root. Nevertheless, a yellowish precipitate, produced in the remaining portion of the drink, by the addition of hydrosulphuret of potash, inclines the authors of this memoir to think that it might also contain some metallic substance equally venomous, the action of which was added to that of the root of hellebore. Though, according to their own experiment, this concurrence of two poisons does not seem necessary to explain what took place; we likewise think that it is to be regretted that Mr. Ferrary did not think proper to make a chemical analysis of this precipitate, in order to determine its nature.

We shall say, in concluding this article, that one ought to be no less on one's guard against the venomous properties of the fetid hellebore, (*helleborus fœtidus*, L.) than on those of the other species of this genus; it is very common all over France, and known by the name of *pied de gryphon*. It has sometimes been employed as a vermifuge; but only skilful hands should use such formidable weapons.

---

